# Genetic Contribution of Ningmai 9 Wheat to Its Derivatives Evaluated by Using SNP Markers

**DOI:** 10.1155/2016/3602986

**Published:** 2016-08-29

**Authors:** Peng Jiang, Ping-Ping Zhang, Xu Zhang, Hong-Xiang Ma

**Affiliations:** Provincial Key Lab for Agrobiology, Jiangsu Academy of Agricultural Sciences/Jiangsu Collaborative Innovation Center for Modern Crop Production, 50 Zhongling Street, Nanjing, Jiangsu 210014, China

## Abstract

Founder parent usually plays an important role in wheat breeding. Ningmai 9 is a soft wheat variety with good performance in yield, quality, and resistance to wheat disease. Therefore it serves as an important commercial variety and founder parent in middle and lower Yangtze River of China. To date, 20 new cultivars have been developed from Ningmai 9 and released to wheat production in the last 10 years. In this study, the 90K iSELECT ILLUMINA chip was used to analyze the genotype of Ningmai 9 and its 17 derivatives. The genetic similarity coefficients between Ningmai 9 and its derivatives were more than 0.7 except for Yangfumai 4. Neighbor-Joining analysis showed that Yangfumai 4 had the largest genetic distance from Ningmai 9 in all derivatives. There was a great difference for the same allele ratio in either derivatives or chromosomes, though the average values of the same allele ratio in genomes A, B, and D were close to each other. The phenotypic difference in Ningmai 9, Ningmai 13, and Yangfumai 4 was consistent with their difference in genetic background by comparing previous reported QTLs. Some hot chromosome regions were found and might be used for marker assisted selection in wheat breeding.

## 1. Introduction

Founder parents are varieties which were used to produce derivatives with desired comprehensive traits. The derivatives are more similar to founder parent rather than the mean of two parents in such traits in breeding practice, as founder parents usually have excellent comprehensive traits and high general combining ability in genetics. Genomics research found that founder parents usually contributed multiple favorable alleles to derivatives. Russell et al. (2000) analyzed the genetic diversity in 19 barley founder parents and their derivatives and found that the founder parents contributed 72% genetic variation to the derivatives [1]. Bharadwaj et al. (2002) reported that 10 Indian parents gave a genetic contribution of 72.6% to 66 soybean varieties in 1968–2000 [2]. It is very valuable to dissect the transmission efficiency and frequency of the genetic information from founder parents to their derivatives for the further utilization of founder parents in breeding.

Wheat is one of the three major grain crops in the world, occupying a very important position in food security. Survey of genetic contribution of founder parent to its derivatives is important in wheat breeding. Zhuang (2003) proposed 16 founder parents which were widely used in China. Among them, a few varieties, such as Aimengniu, Xiaoyan 6, and Zhou 8425B, presented similar properties as founder parent [3]. Li et al. (2009) used 344 polymorphic simple sequence repeat (SSR) markers to screen Orofen and its 23 derivatives, and 21 SSR loci were found constant in Orofen and the derivatives [4]. “Triumph/Yanda 1817” was a core cross in northern China, and the contributions of Triumph and Yanda 1817 to their 38 derivatives were 43.6% and 26.8%, respectively [5]. Aimengniu was derived from the cross “Aifeng3//Mengxian201/Neuzucht”, and the three parents gave an average contribution of 43.2%, 35.4%, and 52.2% to its 41 derivatives [6]. All the founder parents showed a high transfer capacity of genetic information to their derivatives. However, most researches to identify the genetic contribution from parent to its derivatives in genomic level were mainly based on simple sequence repeat (SSR) markers [1, 4, 5, 7]. SSR markers were widely used in marker assisted selection, but the smaller amount of polymorphism limited their application in genetic contribution analysis. Single nucleotide polymorphism (SNP) is the most popular molecular marker at present for its genetic stability, large amount, and wide spread in genome. Some high-density gene chips based on SNP have been developed and widely used in genetic analysis of wheat, maize, rice, and so forth [8–10].

Ningmai 9 is a soft wheat variety widely used in wheat production in the middle to low valley area of Yangtze River in China. As a parent, Ningmai 9 produced 20 cultivars released in the last 10 years. Two varieties derived from Ningmai 9, Ningmai 13, and Yangfumai 4 have been the major varieties in the middle to low valley area of Yangtze River in China. Either of them was planted, more than 200,000 ha each year. In this study, the 90K iSELECT ILLUMINA chip was used to screen Ningmai 9 and its 17 derivatives to show an accurate genetic contribution of Ningmai 9 to its derivatives, especially to the major varieties Ningmai 13 and Yangfumai 4 (Figure 2). The results may provide a basis for the further application of Ningmai 9 in wheat breeding.

## 2. Materials and Methods

### 2.1. Plant Material and DNA Extraction

Ningmai 9 and its 17 derivatives were used as the experimental materials in this study (Table 1). The DNA was extracted from fresh leaf tissue (100 mg) following a standard CTAB protocol and then diluted to 50 ng/*μ*L for genotyping.

### 2.2. SNP Assay

Genotyping procedure for the 90K iSELECT ILLUMINA chip was performed as described by Wang et al. (2014) [11] at the USDA Genotyping Center. Genotypic clusters for every SNP were determined using the manual option of Genome Studio version 1.9.4 with the polyploid clustering version 1.0.0 (Illumina), on the basis of the data from all of the described genotypes. A total of 81587 SNPs including BS, Bob White, CAP, and D contig were obtained.

### 2.3. Data Analysis

6904 SNPs out of 81587 SNPs were selected according to 3 filtering conditions: there was no missing data, there was difference among the materials, and there was an ensured position on the chromosome. The 6904 screened SNPs were used for further analysis. Excel 2007 was used for data processing and statistic analyzing. NTSYSpc version 2.10t was used to calculate genetic similarity coefficients (GSCs), and Neighbor-Joining tree was constructed by MEGA 6.0 [12].

## 3. Results

### 3.1. Genetic Relationship among Ningmai 9 and Its Derivatives

**Figure 1:**
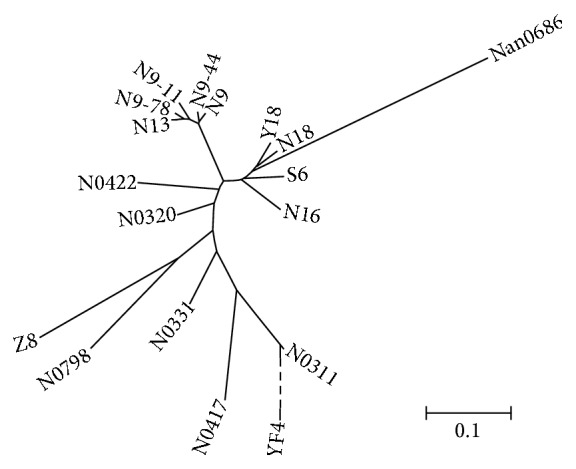
Dendrogram of Ningmai 9 and its derivatives based on Neighbor-Joining analysis. The genetic distance of Yangfumai 4 was very large and broken down with dashed line.

**Figure 2:**
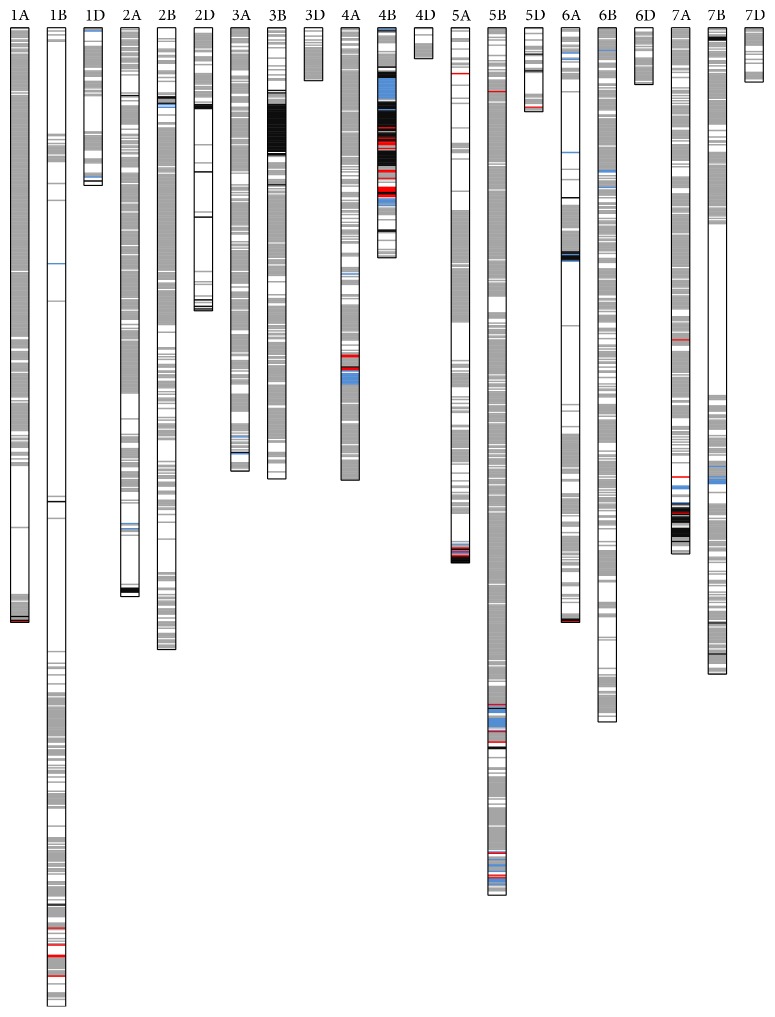
Genetic contribution of Ningmai 9 to Ningmai 13 and Yangfumai 4. White regions are identical sections in Ningmai 9, Ningmai 13, and Yangfumai 4; red regions are different sections among Ningmai 9, Ningmai 13, and Yangfumai 4; blue regions represent different section of Ningmai 13, and gray regions represent different section of Yangfumai 4; and black regions are identical sections between Ningmai 13 and Yangfumai 4, but different from Ningmai 9.

All the genetic similarity coefficients (GSCs) between Ningmai 9 and its derivatives were more than 0.7 except for Yangfumai 4 (Table 2), indicating that Ningmai 9 had a high rate of genetic transmission. Ning 9-44 had the highest GSC (0.98) with Ningmai 9, and the lowest GSC was observed between Nannong 0686 and Yangfumai 4 (0.36). Neighbor-Joining analysis presented similar results (Figure 1). Ningmai 9 and Ning 9-44 clustered together. Yangfumai 4 was clustered at the largest genetic distance. The largest genetic distance was between Nannong 0686 and Yangfumai 4. Neighbor-Joining analysis also reflected the pedigrees to some extent. Ningmai 13, Ning 9-78, Ning 9-44, and Ning 9-11 were all directly selected from Ningmai 9, and they clustered together. Both Yangmai 18 and Ningmai 18 obtained genetic information from Ningmai 9 and Yangmai 158 (Yangmai 10 was derived from Yangmai 158), and Ningmai 16 and Shengxuan 6 were both derived from the cross Ningmai 8/Ningmai 9. Yangfumai 4 was the farthest from the other materials since it was a ^60^Co-*γ* radiated mutant from the hybrid seeds of the cross Ningmai 8/Ningmai 9.

### 3.2. Distribution of Genetic Information of Ningmai 9 on 21 Chromosomes of Its Derivatives

The means of SNPs on each chromosome in genomes A, B, and D were 411, 496, and 80, respectively. There was great difference for the same allele ratios (SAR) in either derivatives or chromosomes. Taking chromosome 1A as an example, SAR between Ningmai 9 and Ning 9-78 reached 100%, while SAR was only 28% between Ningmai 9 and Yangfumai 4 (Table 3). The average values of SAR on different chromosomes were different, which ranged from 62% on chromosome 4D to 91% on chromosome 6D. The SAR values of 18 chromosomes in all 21 chromosomes ranged from 70% to 90%; however, the average values of SAR in genomes A, B, and D were close to each other.

### 3.3. Genetic Contribution of Ningmai 9 to Ningmai 13 and Yangfumai 4

Ningmai 13 and Yangfumai 4 were both derivatives of Ningmai 9, but there was great difference in the genetic background, and the GSC between them was only 0.45. Ningmai 13 was more similar to Ningmai 9 than Yangfumai 4 was. Few variations were observed on chromosomes of Ningmai 13, especially on chromosomes 3D, 4D, 6D, and 7D, which were fully consistent with Ningmai 9, while variable regions were over 50% on most chromosomes of Yangfumai 4 in comparison with the alleles in Ningmai 9. The regions where Ningmai 13 and Yangfumai 4 shared the same variation were observed on chromosomes 3B, 4B, and 5A, while the regions with different variations between Ningmai 13 and Yangfumai 4 were presented on chromosomes 1A, 1B, and 4A.

## 4. Discussion

Ningmai 9 is a soft wheat variety with high yield, wide adaptation, moderate resistance to Fusarium head blight and sharp eye spot, and high resistance to wheat yellow mosaic virus and serves as important parent in middle and lower valley of Yangtze River in China. Twenty new wheat cultivars have been developed from Ningmai 9 in the last 10 years. Founder parents easily produce varieties because of their excellent comprehensive characters and high genetic combining ability [3]. From the genomic point of view, the founder parent has favorable alleles associated with comprehensive traits. Su et al. (2006) identified 39 QTLs related with number of tillers and phosphorus absorption efficiency with a double haploid population from Lovrin 10/Chinese Spring, and 15 of them were from Lovrin 10 [13]; Christopher et al. (2007) discovered a few high frequency alleles related with some important traits with a wheat pedigree based on Cook and a barley pedigree based on crosses between Triumph and Koru [14]; Ma et al. (2007) used a double haploid population from Nanda 2419/Wangshuibai to identify a few QTLs controlling ear length and ear density from Nanda 2419 [15]. Yao et al. (2012) identified the high combining ability of Ningmai 9 in yield, quality, and resistance and the successful application in wheat breeding [16]. In this study, we analyzed the genotype of Ningmai 9 and its derivatives by using 90K iSELECT ILLUMINA chip and found that the GSCs between Ningmai 9 and its derivatives were mostly over 0.7 except for Yangfumai 4, which was significantly higher than its theoretical expectation, indicating a high rate of genetic transmission in Ningmai 9.

Ningmai 16, Shengxuan 6, Ning 0311, Ning 0417, and Yangfumai 4 were derived from the same cross of Ningmai 8/Ningmai 9. Theoretically, the varieties from the same cross combination may have high GSCs and close genetic distance in the dendrogram based on Neighbor-Joining analysis. Ningmai 16 and Shengxuan 6 confirmed the theory, whereas other derivatives from the same cross were relatively far apart, such as Ning 0311 and Ning 0417, which means that the selection by breeders was a crucial factor affecting the genetic background of offspring from the same cross. Yangfumai 4 was a ^60^Co-*γ* radiated mutant of hybrid seeds from the same cross and had the lowest GSC with Ningmai 9, which indicated that radiation was an effective way to create some novel genetic variation.

Backcross can improve the genetic proportion of the recurrent parent significantly. Yangmai 18 and Ningmai 18 experienced 4 and 2 rounds of backcross with Ningmai 9, respectively, and their GSCs with Ningmai 9 were only second to the derivatives directly selected from Ningmai 9.

Ningmai 13 is the most popular soft wheat variety, and Yangfumai 4 is the most popular hard wheat in middle and lower Yangtze River of China now. Ningmai 13 inherited the features of good soft wheat quality, effective tillers, and FHB resistance from Ningmai 9 and improved lodging resistance and thousand kernel weight (TKW). Yangfumai 4 presented a distinct difference from Ningmai 9 and had a hard wheat quality with high to medium gluten content, big ear, and large size kernel. Ningmai 13 and Yangfumai 4 both had high resistance to wheat yellow mosaic virus as Ningmai 9. Ningmai 9 possessed the wheat yellow mosaic virus resistant QTL with the same allele on chromosome 2DL and 3BS as previous reports [17, 18]. Ningmai 13 and Yangfumai 4 had the identical alleles on the chromosome regions with Ningmai 9. For the resistance to Fusarium head blight (FHB), Ningmai 9 had the major QTL* Fhb1*, which was the most important FHB resistant QTL on chromosome 3BS [19]. Ningmai 13 and Yangfumai 4 also had the same chromosome region as in Ningmai 9 and performed the resistance to FHB. Many QTLs controlling grain number per ear, ear weight, and ear length had been reported on chromosomes 2A, 2B, 2D, 4A, 5A, 5D, and 6B [20–22], where most variations were only observed in Yangfumai 4. The QTLs controlling thousand kernel weight were identified on chromosomes 2A, 2D, 3B, 4B, 6A, 7A, and 7B [21, 23], and those controlling lodging resistance were discovered on chromosomes 2A, 2D, 3B, 5A, 5B, and 5D [24–26], on which Ningmai 13 and Yangfumai 4 both presented same alleles. These chromosome regions may contain QTLs associated with thousand kernel weight and lodging resistance. Such hot chromosome regions might be used for marker assisted selection in wheat breeding.

In the study, we validated that Ningmai 9 is a founder parent on genomic level. The derivative varieties inherited a high proportion of the genetic background from Ningmai 9, especially on some hot variation regions associated with desired comprehensive characters.

## Figures and Tables

**Table 1:** Name and pedigree of selected varieties.

Variety (line)	Abbreviations	Pedigree
Ningmai 9	N9	Yangmai 6/Xifeng
Ningmai 13	N13	Direct selection from Ningmai 9
Zhenmai 8	Z8	Yangmai 158/Ningmai 9
Nannong 0686	Nan0686	MV964091/Ningmai 9
Yangmai 18	Y18	Ningmai 9^*∗*^4/3/Yangmai 158^*∗*^6//88-128/Nannong P045
Ningmai 18	N18	Ningmai 9^*∗*^2//Ningmai 9/Yangmai 10
Ning 9-78	N9-78	Direct selection from Ningmai 9
Ning 9-44	N9-44	Direct selection from Ningmai 9
Ning 9-11	N9-11	Direct selection from Ningmai 9
Ningmai 16	N16	Ningmai 8/Ningmai 9
Ning 0311	N0311	Ningmai 8/Ningmai 9
Ning 0320	N0320	Ningmai 8/Ningmai 9
Ning 0331	N0331	Ningmai 8/Ningmai 9
Ning 0417	N0417	Ningmai 8/Ningmai 9
Ning 0422	N0422	Ningmai 8/Ningmai 9
Ning 0798	N0798	Ningmai 9 large/Yangfu 9798//Sumai 6
Yangfumai 4	YF4	Mutant induced by _ _^60^Co-*γ* radiation from Ningmai 8/Ningmai 9
Shengxuan 6	S6	Ningmai 8/Ningmai 9

**Table 2:** Genetic similarity coefficient among N9 and its derivatives.

	N9	N13	Z8	Nan0686	Y18	N18	N9-78	N9-44	N9-11	N16	N0311	N0320	N0331	N0417	N0422	N0798	YF4
N13	0.96																
Z8	0.78	0.77															
Nan0686	0.71	0.69	0.61														
Y18	0.86	0.84	0.73	0.75													
N18	0.89	0.87	0.72	0.75	0.94												
N9-78	0.96	0.97	0.77	0.70	0.85	0.87											
N9-44	0.98	0.96	0.78	0.71	0.86	0.89	0.96										
N9-11	0.95	0.95	0.77	0.69	0.85	0.87	0.96	0.95									
N16	0.85	0.83	0.73	0.74	0.89	0.89	0.83	0.84	0.82								
N0311	0.76	0.77	0.77	0.61	0.76	0.75	0.76	0.77	0.76	0.76							
N0320	0.85	0.83	0.75	0.72	0.87	0.88	0.83	0.84	0.83	0.89	0.80						
N0331	0.80	0.78	0.74	0.66	0.85	0.84	0.78	0.80	0.79	0.83	0.77	0.84					
N0417	0.73	0.74	0.69	0.60	0.73	0.73	0.73	0.73	0.73	0.75	0.75	0.79	0.78				
N0422	0.82	0.82	0.71	0.70	0.83	0.83	0.82	0.83	0.82	0.86	0.78	0.85	0.79	0.75			
N0798	0.78	0.78	0.75	0.67	0.73	0.73	0.78	0.78	0.77	0.78	0.71	0.79	0.75	0.72	0.75		
YF4	0.43	0.45	0.44	0.36	0.45	0.44	0.44	0.43	0.44	0.44	0.51	0.47	0.48	0.47	0.46	0.43	
S6	0.86	0.84	0.73	0.74	0.91	0.91	0.84	0.86	0.84	0.90	0.75	0.86	0.83	0.73	0.87	0.74	0.45

**Table 3:** Same allele ratios (SAR) between Ningmai 9 and its derivatives on 21 chromosomes (%).

	SNPs	N13	Z8	Nan0686	Y18	N18	N9-78	N9-44	N9-11	N16	N0311	N0320	N0331	N0417	N0422	N0798	YF4	S6	Mean
1A	460	99	57	76	93	99	100	100	99	76	58	90	80	86	69	99	28	76	82
2A	440	98	80	78	98	93	99	99	99	98	79	98	88	79	80	83	40	80	87
3A	343	99	50	92	95	92	95	96	97	90	59	72	58	63	95	50	28	95	78
4A	350	95	90	67	93	92	99	100	99	93	86	93	75	47	79	92	27	90	83
5A	414	97	94	71	79	80	97	98	96	85	76	79	79	83	88	90	54	88	84
6A	460	97	97	27	76	96	97	97	97	96	94	95	95	95	95	95	66	95	89
7A	407	94	81	66	58	64	93	100	87	62	56	61	55	50	60	85	29	55	68
Mean	411	97	78	68	85	88	97	98	96	86	73	84	76	72	81	85	39	83	82

1B	757	99	98	87	90	89	98	99	98	87	86	85	89	85	86	87	77	89	90
2B	481	99	69	72	78	89	97	97	97	76	68	73	77	60	74	70	43	73	77
3B	349	88	78	59	76	83	87	89	87	80	79	89	78	69	79	72	29	86	77
4B	178	51	49	87	72	90	52	90	37	88	54	87	62	31	61	30	36	88	63
5B	671	95	80	56	82	82	95	96	96	77	73	80	83	80	80	66	20	83	78
6B	537	99	76	90	95	98	99	100	99	97	92	96	78	81	95	89	39	97	89
7B	500	98	51	70	98	98	98	98	98	87	80	86	94	75	95	74	49	98	85
Mean	496	90	71	74	84	90	89	96	87	85	76	85	80	69	81	70	42	88	80

1D	122	98	78	55	81	81	100	99	98	68	70	68	64	69	66	64	53	81	76
2D	219	96	90	74	95	93	98	95	97	97	91	94	89	55	97	39	75	97	86
3D	41	100	98	78	71	93	78	100	76	63	73	73	71	63	76	83	27	71	76
4D	24	100	54	29	54	54	100	100	58	54	54	58	54	50	54	71	46	54	62
5D	65	95	85	48	94	80	95	97	95	85	83	94	94	75	82	91	60	95	85
6D	44	100	98	95	95	98	100	100	100	93	91	91	98	77	95	89	30	93	91
7D	42	100	86	67	86	90	93	100	93	71	71		74	76	79	93	45	90	82
Mean	80	98	84	64	82	84	95	99	88	76	76	81	78	67	78	76	48	83	80

Overall mean	329	95	78	69	84	87	94	98	91	82	75	83	78	69	80	77	43	85	
